# A novel piecewise-linear method for detecting associations between variables

**DOI:** 10.1371/journal.pone.0290280

**Published:** 2023-08-24

**Authors:** Panru Wang, Junying Zhang

**Affiliations:** School of Computer Science and Technology, Xidian University, Xi’an, Shaanxi, China; Wuhan University of Technology, CHINA

## Abstract

Detecting the association between two variables is necessary and meaningful in the era of big data. There are many measures to detect the association between them, some detect linear association, e.g., simple and fast Pearson correlation coefficient, and others detect nonlinear association, e.g., computationally expensive and imprecise maximal information coefficient (MIC). In our study, we proposed a novel maximal association coefficient (MAC) based on the idea that any nonlinear association can be considered to be composed of some piecewise-linear ones, which detects linear or nonlinear association between two variables through Pearson coefficient. We conduct experiments on some simulation data, with the results show that the MAC has both generality and equitability. In addition, we also apply MAC method to two real datasets, the major-league baseball dataset from Baseball Prospectus and dataset of credit card clients’ default, to detect the association strength of pairs of variables in these two datasets respectively. The experimental results show that the MAC can be used to detect the association between two variables, and it is computationally inexpensive and precise than MIC, which may be potentially important for follow-up data analysis and the conclusion of data analysis in the future.

## 1 Introduction

There are various linear or nonlinear associations [1–7] between two variables in the big data era. Detecting the association strength between them is necessary and meaningful for future data analysis [8–11]. Linear association between two variables can be detected through existing methods, however, nonlinear association cannot be detected well by using these existing methods. How to accurately detect the association between two variables is an urgent problem to be solved.

The key indicators used to detect the association between two variables are Pearson coefficient, Spearman coefficient, Kendall coefficient, mutual information and distance correlation coefficient. They can detect the association strength between them, but there are also limitations. Galton [12] first proposed the concept of regression and applied the letter “*r*” to express the degree of correlation, however, he did not realize the concept of negative correlation. Subsequently, Pearson [13] proposed Pearson linear coefficient which is the quotient of covariance and standard deviation between two variables. The Pearson coefficient can be used for detecting the association between two variables, where the association is only statistically linear related. Therefore, Spearman [14] proposed Spearman coefficient on the basic of Pearson coefficient, which can detect linear or nonlinear associations between two variables, but these associations are monotonous. As time went by, more and more methods have been proposed to detect the association between two variables. Kendall raised Kendall coefficient [15] also called Harmony coefficient, but the data must be sorted out by the method of rating. Whereafter, Shannon [16] proposed mutual information [17, 18], which is difficult to calculate because it involves probability density. In 2007, Székely [19] proposed a new statistical correlation method, distance correlation coefficient, which made improvement in the Pearson coefficient’s shortcoming. If there is a nonlinear association between two variables, even if the value of Pearson coefficient is 0, we can’t arbitrarily think that there is no association between them; but if the value of distance correlation coefficient is 0, we can directly think there is no association between them without further analysis. Broadly speaking, these indicators, Pearson coefficient, Spearman coefficient, Kendall coefficient, mutual information, and distance correlation, all can be used to detect the association between two variables. However, these measures have some shortcomings: Pearson coefficient only detects linear association, Spearman coefficient is low precision, Kendall coefficient requires ordered variables, mutual information is difficult to calculate, the distance correlation coefficient is not necessarily 0 when variables are independent.

There are various associations between two variables, which may be some complex nonlinear associations, and may not even be expressed by mathematical functions. In modern times, many measures have been proposed to detect the association between them. Wang et al in 2011 proposed a new measure, R correlation coefficient, to detect linear or simple nonlinear relationship between two variables [20]. The R correlation coefficient is based on the mathematical statistics, and only one simple example is used to prove this measure, which is lack of experimental proof. Meanwhile, Reshef [21] et al proposed a widely used measure, maximal information coefficient (MIC), which can detect extensive correlation relationships such as linear, exponential, periodic, even all functional relationships (a superposition of functions—are not well modeled by a function), but it has high computational complexity. Next, Wijayatunga in 2016 proposed a generalized Pearson coefficient [22] and argued that it can detect any nonlinear dependence if a suitable distance metric was used and all possible maximal dependences were considered, however, this method mainly focuses on discrete variables. Soon afterwards, a new measure [23], G-squared, was proposed in 2017 based on a piecewise-linear regression method, which detects whether two univariate random variables are independent and their association strength. Nevertheless, it is hard to estimate the G-squared and needs to satisfy some regularity conditions. Moreover, when the value of G-squared is zero, it does not mean that these two variables are independent. Although the above measures can be used to detect the association strength between two variables, they have following limitations respectively: R coefficient detects simple nonlinear association and lacks more experimental proof, MIC has high computational complexity, generalized Pearson coefficient mainly focus on discrete variables, and the value of G-Squared is 0 which does not mean that variables are independent.

In this paper, we proposed a new measure, maximal association coefficient (abbreviated as MAC), to detect the association strength between two variables. The MAC is to utilize the piecewise-linear idea to detect the association strength between two variables by Pearson coefficient, including linear or nonlinear associations. The remainder of the paper is organized as follows. In section 2, the detailed description of MAC method is shown. In section 3, the generality and equitability of MAC are verified through two simulation experiments. In section 4, the MAC method is applied to two real datasets to further illustrate that it can detect the association strength between two variables, and the results prove that its performance is comparably or better than MIC. In the end, discussions and conclusions are shown in sections 5 and 6 respectively.

## 2 Proposed MAC measure

**Fig 1:**
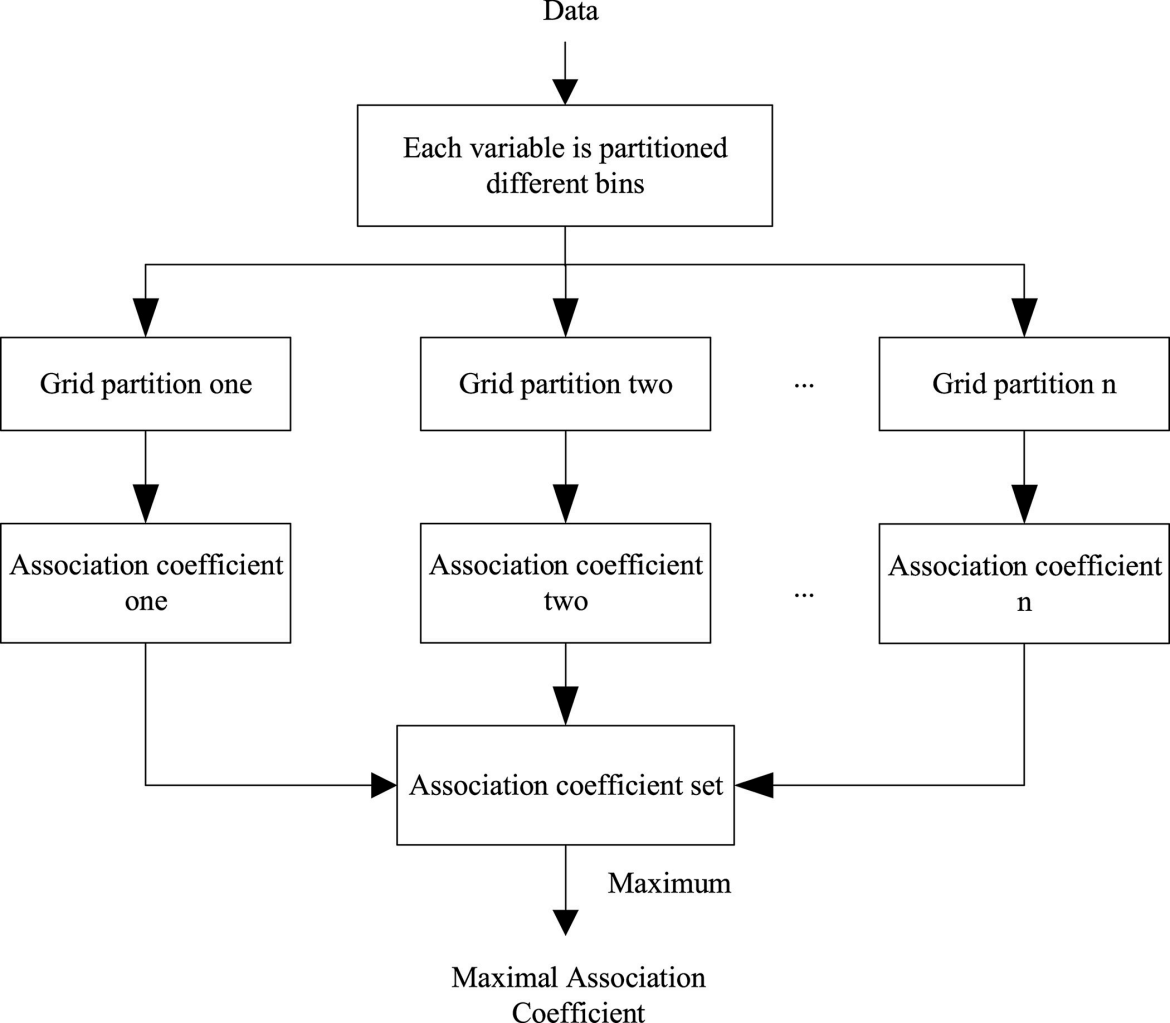
The overview of the proposed MAC measure.

Since the association between two variables can be nonlinear, thus dividing the nonlinear association into some piecewise-linear ones is a way for using linear correlation coefficient to detect nonlinear association between them. In this way, the problem of detecting nonlinear association between two variables is transformed into the problem of detecting multiple simple linear associations. The piecewise-linear can be achieved by partitioning method. The maximal association coefficient is proposed based on partition and linear correlation coefficient, which is summarized in Fig 1. Now, we face two problems. 1) How to divide variables? 2) How to measure linear association?

**Fig 2:**
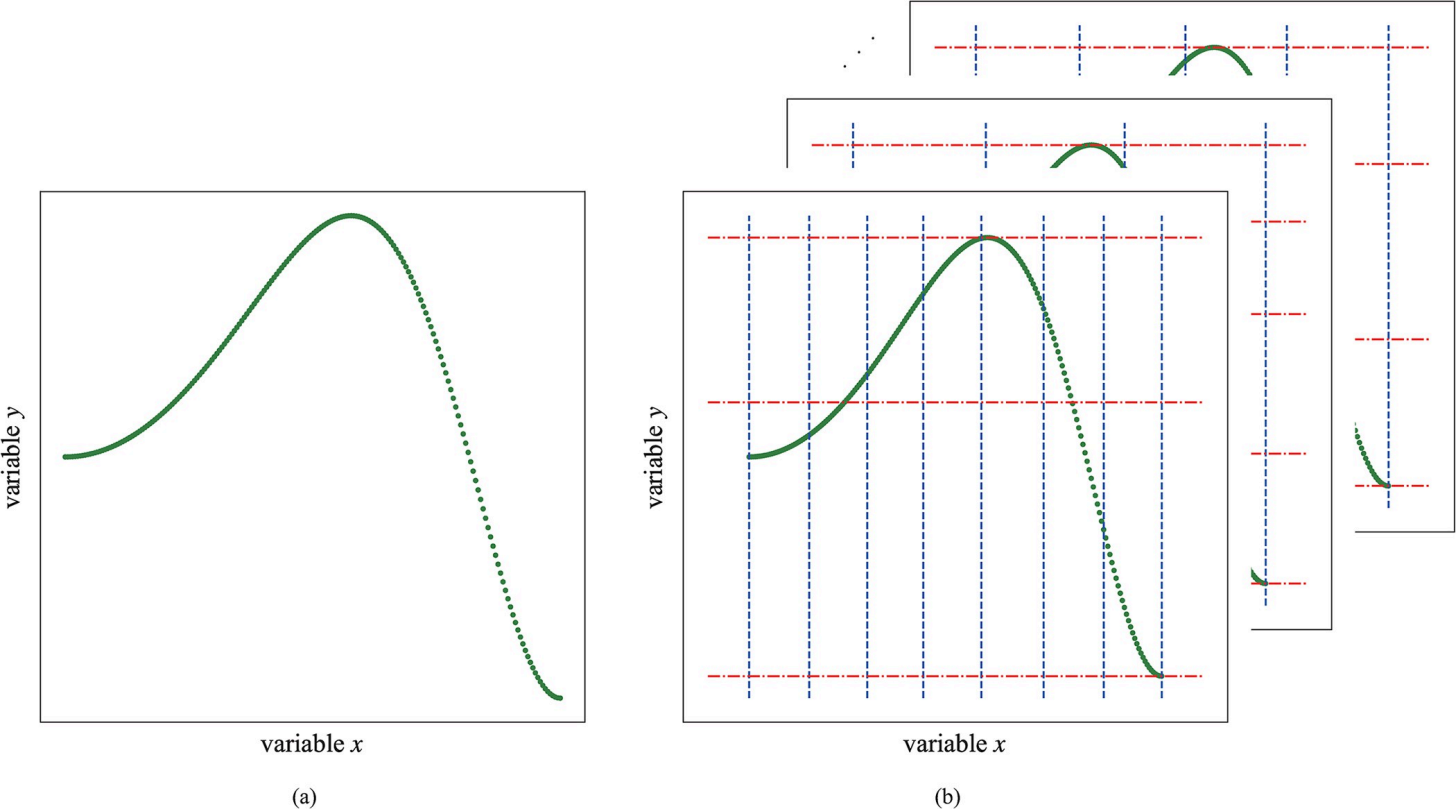
A schematic diagram of grid partition between two variables. (a) This figure is a two-dimensional scatter plot between two variables. (b) There are some different grid partitions between them.

Any nonlinear association between two variables can be considered to be composed of some piecewise-linear ones. However, no one knows where the breakpoint for connecting two piecewise-linear ones is, which is why random partition is suggested. The following problem is that there are an infinite number of partitions, which makes it impossible to detect the association strength between two variables. Clustering techniques becomes one of the options used to achieve the partition. Any clustering technique can be used to divide variables, among which the simple and effective K-means clustering is used here. The K-means method is utilized to divide each variable space into different bins, where K value determines the number of bins of each variable, and then the grid partition between two variables can be obtained. All data can be divided into different grids, and a schematic diagram is shown in Fig 2. We divide the data into different grids, the association coefficients of data in some grids are likely to be larger than that of the whole data. Nevertheless, we employ the combination of the association coefficients from all grids instead of one grid to reveal the association of the whole data. If the association coefficients of only minority grids are large, it is unlikely to cause a higher MAC than the real association coefficient.

About how to detect linear association strength of the data in each grid obtained by dividing two variables, any measures can be used. Among them, Pearson coefficient was proposed early and easy to compute, and until now, it is still the most widely used correlation coefficient index. Thus, Pearson coefficient, is used to detect the association strength of the data in each grid after obtaining the grid partition between two variables. The Pearson coefficient is between [–1,1], which can detect whether the association between two variables is positive correlation, uncorrelation or negative correlation. After obtaining the Pearson coefficient of data in each grid, the weighted sum obtained by directly using the Pearson coefficient offsets by the positive and negative values, which causes the result of weighted sum cannot reflect the association strength between two variables. We aim to detect whether the association between variables is correlated and the degree of correlation. Therefore, we take the absolute value of the Pearson coefficient to reveal the association, and then if the coefficient is 0 that indicates uncorrelation, 1 indicates perfect correlation, and 0 to 1 indicates different degrees of correlation. As a result, the weighted sum obtained by using the absolute value of the Pearson coefficient can well reflect the association between two variables.

For the grid partition between two variables, it is necessary to set a maximum number of grids (MG) to avoid the infinite grids caused by the method of partition. The maximum number of grids (MG) is MG = max{4, *n*^*α*^}, where *n* is data size and α is a hyper-parameter and α belongs to [0, 1]. For variable *x* and variable *y*, they are divided into *s* and *t* bins, respectively, where *t* is equal to *MG*/*s*. The data is divided into any *s*-by-*t* grid, where *s* belongs to [2, MG/2], and thus many different forms of grid partition between two variables can be obtained. Under each grid partition, an association coefficient (AC) between variables *x* and *y* is calculated based on Eq 1.


$$AC{\left(x,y\right)}_{\left(s,t\right)}=\sum_{i}w_{i}|p_{i}|$$ (1)

In Eq 1, *i* is the i-th grid containing data, *w*_*i*_ is the weight of the i-th grid, and |*p*_*i*_| is the absolute value of Pearson coefficient of data in the i-th grid. The *w*_*i*_ can be obtained according to Eq 2.


$$w_{i}=\frac{s_{i}}{\sum_{j}s_{j}}$$ (2)

In Eq 2, *s*_*i*_ is the area of the i-th grid containing data, and ∑_*j*_*s*_*j*_ is the sum of areas of all these grids. Moreover, in Eq 2, the weight *w*_*i*_ is the normalization of the area, so that the sum of weights is 1, i.e., the ∑_*i*_*w*_*i*_ is 1.

The association coefficient under each grid partition can be obtained by Eq 1, in which the maximum value of these association coefficients is the maximal association coefficient (MAC), as shown in Eq 3.


$$MAC(x,y)=\underset{s,t}{\max}\{AC{\left(x,y\right)}_{\left(s,t\right)}|s*t=MG\}$$ (3)

The description of **MAC algorithm** is described in the following. The inputs of MAC algorithm are a dataset *D* with data size *n*, and a hyper-parameter α in which the hyper-parameter α determines the maximum number of grids (MG). The output of MAC algorithm is maximal association coefficient, i.e., MAC, between two variables.

**Table:** 

**MAC algorithm**
**Input**: Dataset *D* with data size *n*; a hyper-parameter α and α∈[0, 1]
**Output**: maximal association coefficient (MAC) between two variables
1: *s*←2, *t*←2
2: while *s*×*t* = *MG* do
3: the association coefficient obtained under each grid partition
4: end while
5: the MAC between variable *x* and variable *y*
6: return MAC

The calculation steps of MAC are shown as follows:

**Step 1**. There are two variables labeled as *x* and *y*, respectively. Then, K-means method is used for dividing x-values into *s* bins and y-values into *t* bins, in this way, any *s*-by-*t* grid partition can be obtained.

**Step 2**. The area of each grid containing data, and the Pearson coefficient of data in this grid is calculated, respectively. The ratio of the area of each grid containing data to the sum of the areas of all these grids is used as the weight of the corresponding grid.

**Step 3**. The weighted sum of the absolute value of the Pearson coefficient of the data in each grid and the weight of this grid is called association coefficient between two variables under this partition.

**Step 4**. Repeat step 1 to step 3, many association coefficients can be obtained under the constraint of the maximum number of grids (MG). The maximum value of these association coefficients is the maximal association coefficient between two variables.

## 3 Simulation experiment and result analysis

In section 2, the MAC method is proposed and described in detail for detecting the association strength between two variables. If a measure can detect the association strength under extensive relationships with enough sample size, this measure is of generality; If a measure can give similar scores to different relationship types with the same noise, this measure is of equitability. We assume that MAC should be of generality and equitability. Thus, we verify and analyze whether the MAC is generality and equitability through a series of simulation experiments, in this section 3.

In this section, the hardware used is Intel (R) Core (TM) i7-10510U CPU @ 1.80GHz 2.30GHz, 8.00GB RAM and the software is Anaconda Python 3.7.

### 3.1 Data type

There are 10 different relationship types used in this section are shown in Table 1, where the corresponding relational expressions are also shown in the table. The data *x*_*i*_ (in which *i* is 1, 2, …, n) in the variable *X* is generated in the domain [0, 1], and the data *y*_*i*_ (in which *i* is 1, 2, …, n) in the variable *Y* is obtained according to the relational formula given in Table 1. These relationship types are used to verify the generality and equitability of the MAC.

**Table 1:** Multiple different relationship types between two variables.

Relationship type	Relational expression (where *X*∈[0, 1])
Linear	*Y = 2X + 1*
Exponential	*Y = 4* ^*(X-o*.*5)*^
Triangle composite function	*Y = sin(0*.*5πX*^*2*^*)*
Sine	*Y = sin(2πX)*
Parabolic	*Y = (X-0*.*5)*^*2*^ *+ 4*
Periodic + Linear	*Y = sin(10πX) + X*
Sinusoidal (Fourier frequency)	*Y = sin(16πX)*
Sinusoidal (non-Fourier frequency)	*Y = sin(13πX)*
Sinusoidal (varying frequency)	*Y = sin(7πX(1+X))*
Random	Random Number Generator

### 3.2 Generality

**Fig 3:**
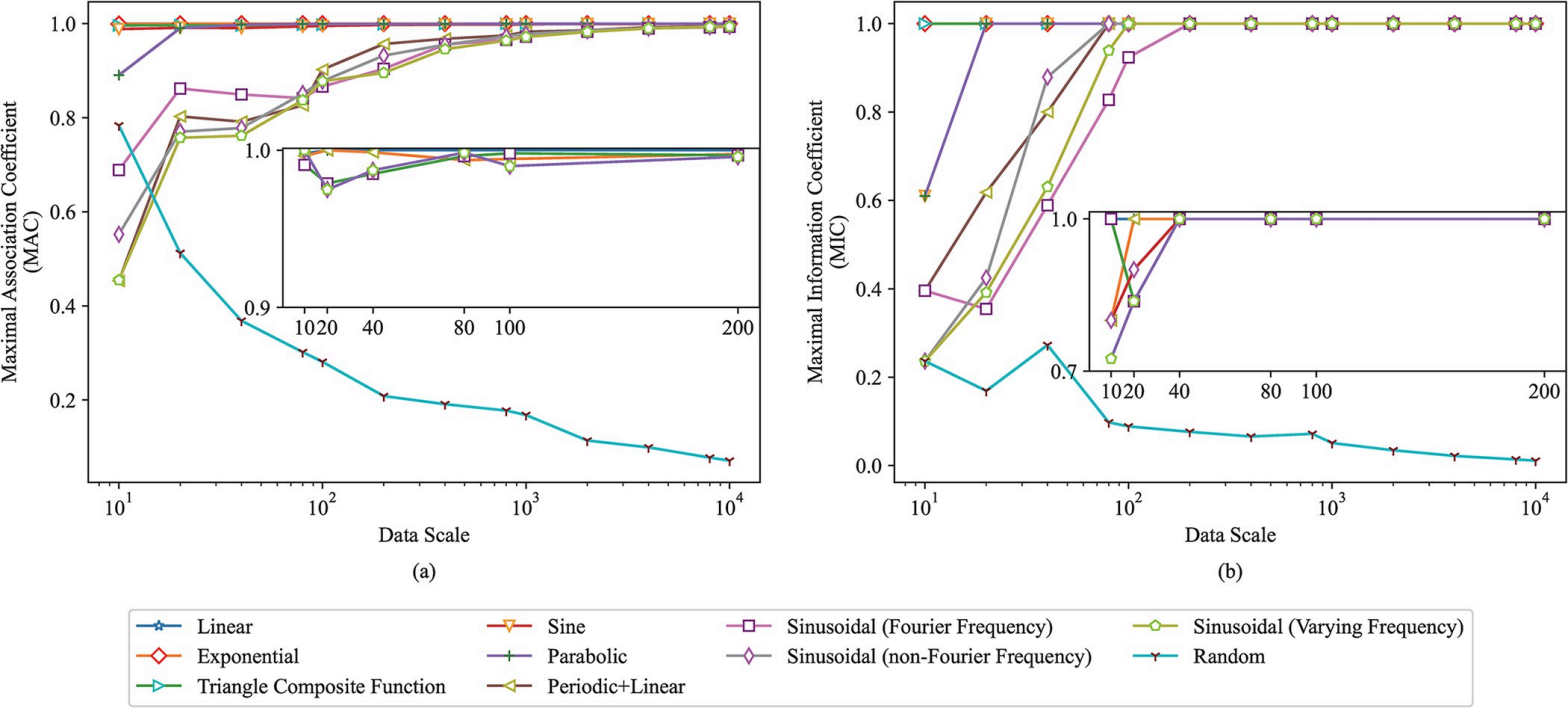
The MACs and MICs between two variables with different data scales under each relationship type. (a) The MAC between variables. (b) The MIC between variables.

Under each relationship type in Table 1, datasets with data sizes *n* of 10, 20, 40, 80, 100, 200, 400, 800, 1000, 2000, 4000, 8000, 10000 are generated, totaling 13 datasets. Under each dataset, the value of the variable *X* is randomly generated in the domain [0, 1], and then the corresponding the value of the variable *Y* is generated according to the corresponding relational expression in Table 1. The first nine types in Table 1 are non-random relationship types, and the tenth type is random relationship types. The data (*x*_*i*_, *y*_*i*_) with different data scales are generated under each relationship type, where *i* = 1, 2,…, *n*, and the MAC between two variables in the data is calculated. In this section, we apply MAC and MIC methods to each dataset under each relationship type, where the hyper-parameter α is empirically set to 0.75 for the first nine relationship types and that is empirically set to 0.45 for the random relationship type. We repeat the MAC and MIC computation for 50 times to obtain 50 MACs and MICs, and then take the average of 50 MACs and MICs as the MAC and MIC of each dataset to reduce the influence of randomness on the experiments. As the data scale increases, the MAC and MIC between two variables under each relationship type are shown in Fig 3A and 3B. The MACs and MICs of different relationship types are represented by these different colored polylines is shown in the legend in Fig 3.

As can be seen from Fig 3A and 3B, for the first four simple relationship types, even if the data scale is small, their association can be accurately detected, that is, the MAC and MIC tends or equals to 1.0. For the latter five complex relationship types, their association strength can be accurately detected in a big data scale, and their MAC and MIC approaches to 1.0. It is noted that there is still a strong association for these five relationship types even in a small data scale, so we try to change the value of hyper-parameter α to observe whether the MAC and MIC methods can detect the association between them. We set the hyper-parameter α in the MAC and MIC methods to 1.0 to detect their association under a small data scale with data size of 10, 20, 40, 100, 200, and their MACs and MICs are shown in an insert figure in Fig 3A and 3B. We can know that MAC method can completely detect their association even if there are only 10 data points, but MIC method requires at least 40 data points except for the parabolic relationship type. Our MAC method is more robust than MIC method for a small data scale. For relationship types with different complexity, the α can also be set to different value when detecting their association strength. Additionally, when the data scale is large (or small), the value of α can be small (or large).

**Fig 4:**
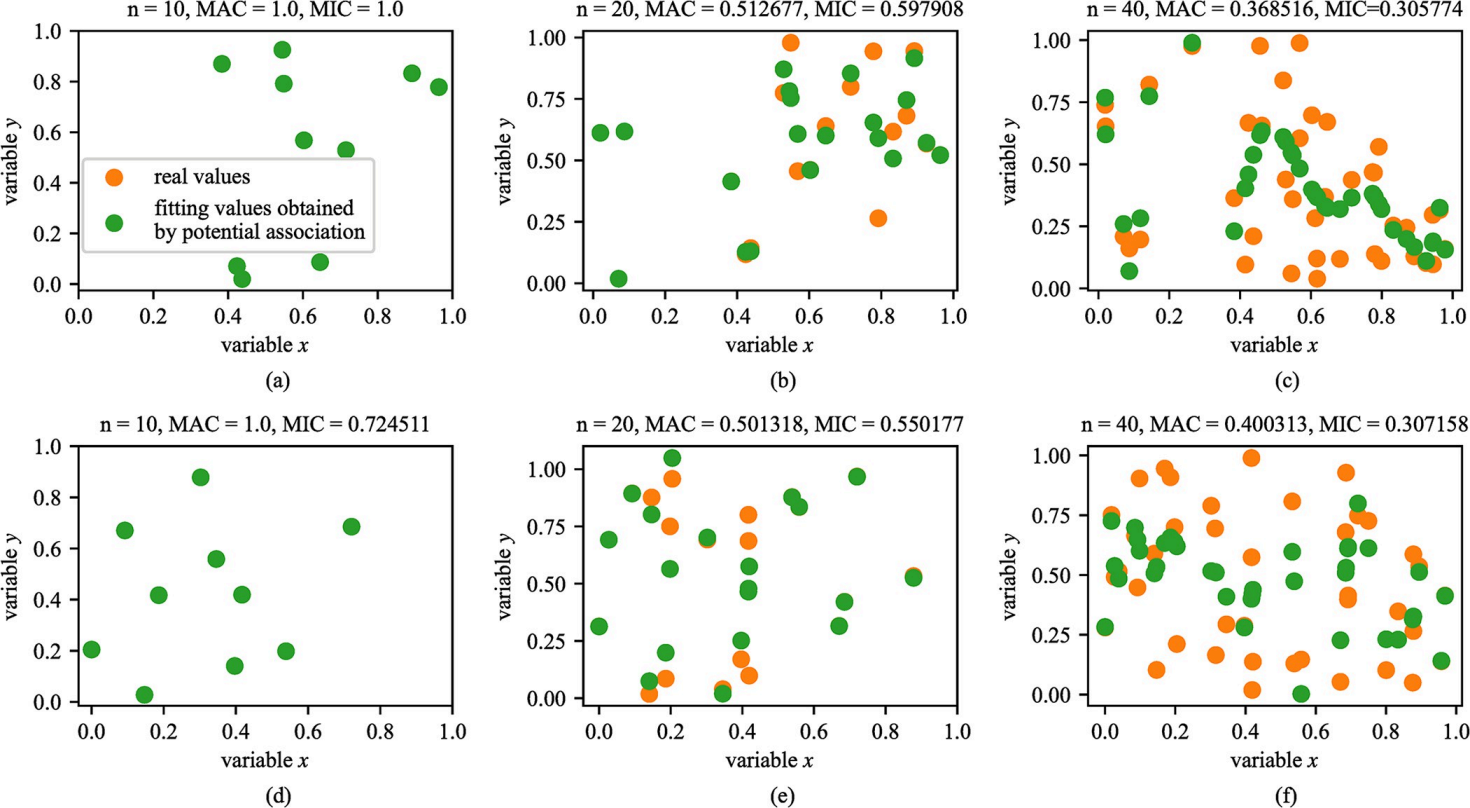
The MACs and MICs between two variables under the random relationship type. The text at the top of each figure reveals the data scale and the MAC and MIC between variables.

When the relationship type between two variables is random, the MAC and MIC generally decrease with the increase of data scale until they tend to zero. Especially, there is an interesting phenomenon for the random relationship type, that is, the MAC is large when the data scale is small. We assume that there is an unknown relationship between variables when the data scale is small. We explain this phenomenon through an experiment, as shown in Fig 4, where n (n = 10, 20, 40) is data size. Mathematically, it can be proven that any function can be expressed in polynomial form. We employ polynomial function to fit the randomly generated data points. When only 10 data points are randomly generated, we can employ a polynomial function to completely fit them, as shown in Fig 4A and 4D. The MACs between variables in Fig 4A and 4D are all 1.0, but the MIC is 1.0 in Fig 4A and not 1.0 in Fig 4D. This may be because our MAC method is more robust than MIC method for a small data scale, which has been mentioned in the above. As the data scale increases, the randomness between variables also increases and the MAC and MIC gradually decreases, as shown in Fig 4B, 4C, 4E and 4F. In summary, the MAC and MIC may be large when data scale is small even in the random relationship type.

Additionally, it can be shown from Fig 3A that the MAC of deterministic relationship type increases as the data scale increases, and the MAC of random relationship type decreases. When the actual data size up to 10000, the MAC between two variables infinitely approaches to 1.0 in addition to the random relationship type. The MAC can be used to detect the association strength of multiple complex relationship types. Therefore, the MAC has good generality.

### 3.3 Equitability

The datasets generated by the first nine relationship types as shown in Table 1 are used to verify the equitability of the MAC. The data of the variable *X* is uniformly generated in the domain [0,1], and then the data of the variable *Y* is obtained according to the relational expression under each relationship type in Table 1, in which the data size *n* is set to 4000. In this way, 9 noise-free datasets are obtained. Uniform vertical noise means that the noise is added to the data of variable *Y*. R^2^ is the squared Pearson coefficient between the perturbed y-values and the true y-values. In other words, 1- R^2^ is the noise added to the true y-values. After that, the noise levels of 10%, 20%, 30%, 40%, 50%, 60%, 70%, 80%, 90%, and 100% were added to the noise-free data set for generating some noise datasets. Considering the noise-free data set, a total of 11 datasets with different noise levels under each relationship type are obtained. The hyper-parameter α determines the maximum number of grids, that is, MG described in section 2. We employ MAC and MIC methods to detect the association between variables. For MIC method, the hyper-parameter α is set to 0.5 for all relationship types. It can be seen from section 3.2 that for different relationship type, the hyper-parameter α in MAC method can be different when detecting their association strength. The simpler the relationship, the smaller the value of α, and the more complex the relationship, the larger the value of α. Thus, for linear, exponential, and triangle composite function, these relationship types are simple and the hyper-parameter α is set to 0.2. For parabolic and sine, these relationship types are slightly more complex and the hyper-parameter α is set to 0.3. For Periodic plus Linear, Sinusoidal (Fourier Frequency), Sinusoidal (non-Fourier Frequency) and Sinusoidal (Varying Frequency), these relationship types are complex and the hyper-parameter α is set to 0.5.

**Fig 5:**
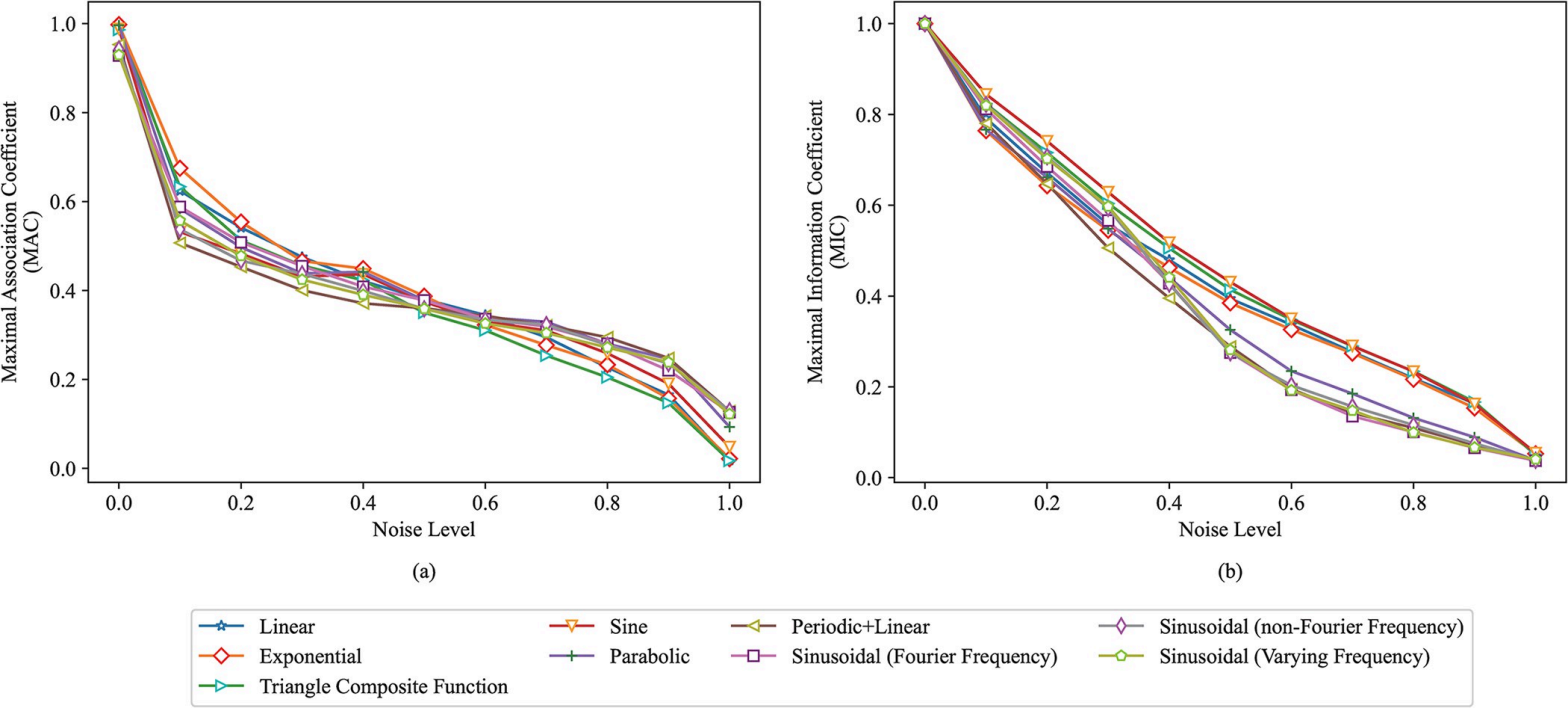
The MACs and MICs between two variables with different noise levels under each relationship type. (a) The MAC between variables. (b) The MIC between variables.

The MAC and MIC methods are applied to each dataset with different noise levels under these nine relationships types, and the MAC and MIC between two variables can be obtained. The MAC and MIC calculation are repeated 50 times, and the average of the 50 MACs and MICs are taken as the MAC and MIC between two variables, as shown in Fig 5. The purpose of taking the average is to reduce the influence of randomness on the experiments. The MACs and MICs of different relationship types are represented by these different colored polylines is shown in the legend in Fig 5. As shown in Fig 5, the MAC and MIC between two variables gradually decreases under the same relationship type as the noise level increases. When the added noise level is the same, the MACs and MICs between two variables under different relationship types are similar. Therefore, the MAC has good equitability. Because there is no baseline association coefficient for data with noise, it is hard to estimate which method is accurate, MAC or MIC methods.

In section 3.2, by exploring the MAC between variables with different data scales under various relationship types, it is found that the MAC between two deterministic variables under the same relationship type gradually increases to 1 or infinitely tends to 1 as the data scale increases, indicating that MAC has good generality. In section 3.3, by exploring the MAC between variables with different noise levels under various relationship types, it is found that the MAC between two variables under the same relationship type decreases as noise level increases, and the MAC between two variables under the different relationship types with the same noise level are basically consistent, indicating that MAC has good equitability. Therefore, the MAC has good generality and equitability.

## 4 Experiments and result analysis on real data

There are various real datasets [24–29], such as performance statistics dataset for the 2008 Major League Baseball season (MLB2008 dataset) [24, 25]; dataset of credit card clients’ default [26]; and so on. In this section, the MAC method is used for two real datasets, MLB2008 dataset and dataset of credit card clients’ default, to verify that MAC method can be used to accurately detect the association strength between two variables. For baseball statistic glossary see: http://www.baseballprospectus.com/glossary/.

### 4.1 Major-league baseball dataset

**Fig 6:**
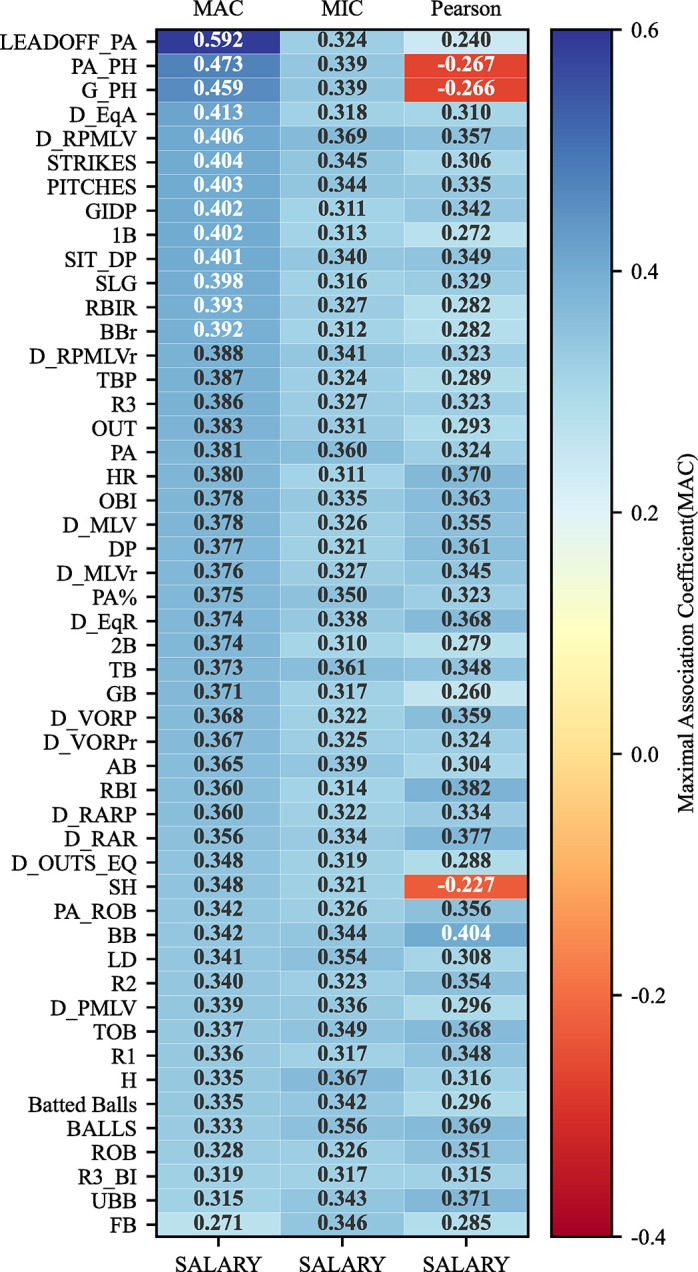
The MAC and MIC and Pearson coefficient of 50 pairs of variables in the MLB2008 dataset.

In this section, the MAC method is used to calculate the maximal association coefficient (MAC) between player’s salary and any one of 50 variables in the MLB2008 dataset which contains 337 instances, to reveal the association strengths between them. The hyper-parameter α in the MAC method is empirically set to 0.45, and a detailed introduction to hyper-parameter α is given in section 2. For proving the ability of the MAC to detect the association strength, MIC and Pearson coefficient were used to detect the association of these 50 pairs of variables in the other two comparative experiments. The MAC, MIC, and Pearson coefficient between player’s salary and any one of 50 variables in the MLB2008 dataset are shown in Fig 6, where the MAC is the average of the results obtained running the MAC algorithm for 50 times and MIC is from the previous work [21]. As shown in Fig 6, the MACs of 50 pairs of variables are represented in MAC column. Similarly, the MICs are shown in MIC column and the Pearson coefficients are shown in the Pearson column.

The evaluation criteria of regression analysis are used to evaluate the difference between MAC and MIC, as shown in Table 2. These evaluation criteria are Mean Squared Error (MSE), Root Mean Squared Error (RMSE), Mean Absolute Error (MAE), where the larger the value, the greater the error. It can be seen from Fig 6 and Table 2 that the difference between MAC and MIC is small, therefore, the MAC can be used to detect the association strength between two variables instead of computationally complicated MIC.

**Table 2:** Differences between the MACs and MICs of 50 pairs of variables in the MLB2008 dataset.

Evaluation criteria	Corresponding value
Mean Squared Error (MSE)	0.00430
Root Mean Squared Error (RMSE)	0.06558
Mean Absolute Error (MAE)	0.04940

### 4.2 Dataset of credit card clients’ default

**Fig 7:**
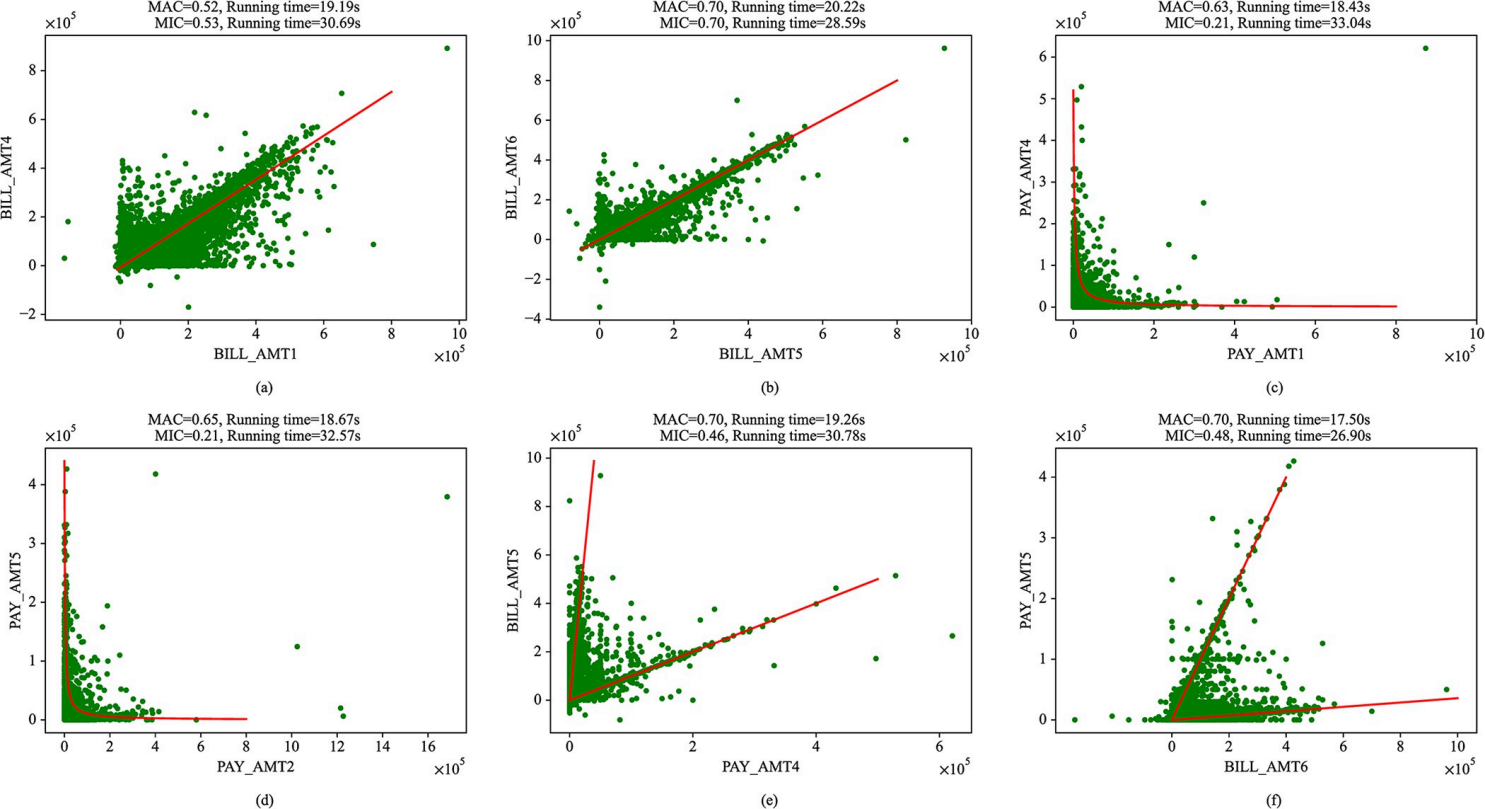
Application of MAC to pairs of variables from the dataset of credit card clients’ default. There are scatter plots of 6 pairs of variables, and the possible associations between variables are also shown in these scatter plots. The text at the top of each figure reveals the corresponding coefficients obtained through MAC and MIC algorithms and the running time of these two algorithms.

To prove MAC method can also be used for large-scale dataset, in this section, this method is applied to a dataset of credit card clients’ default which contains 30000 instances to detect the association between variables. In addition, we also apply MIC method to this dataset for comparing the performance of these two algorithms. We apply these two methods to all pairs of variables from this dataset, where six key associations are chosen and shown in Fig 7. As shown in Fig 7, the green dots represent the data points and the red line represents the possible association between variables. The text at the top of each figure shows the MAC and MIC between variables and these two algorithms’ running time, which are the average of the results obtained after MAC and MIC algorithms are run 50 times. There are linear associations in Fig 7A and 7B, the MAC and MIC between variables are similar but the running time of MAC is lower than MIC. In addition, it can be seen from Fig 7A and 7B that more noise is added to the linear relationship in Fig 7A than in Fig 7B, therefore, the MAC and MIC between variables in Fig 7A are lower than Fig 7B according to the equitability of these two measures. There are similar associations in Fig 7C and 7D, we can see that, on the one hand, the MAC and MIC between variables and their running time are close; on the other hand, the MAC is more precise than MIC and the running time of MAC algorithm is lower than MIC algorithm. Similarly, these phenomena also appear in Fig 7E and 7F. The experimental results show that MAC method is both computationally inexpensive and precise in detecting the association strength compared with MIC method. It proves that MAC method is suitable for big data. It is potentially important to accurately detect the association between variables, such as using these associations for feature selection, etc., which may obtain better conclusion of data analysis.

## 5 Discussions

Both MAC and MIC methods can be used to detect the association strength between two variables, but there are also differences between them. The MAC method is based on piecewise-linear idea, which employs Pearson coefficient to detect the association strength of data in each piecewise-linear for further revealing the association strength between two variables. In addition, K-means in the MAC method is used to divide data into different grid partitions to achieve the piecewise-linear idea. And yet, the MIC method is used to detect the association strength between two variables based on the mutual information, in which mutual information is calculated approximately by the way of grid partition. The MAC is more precise than the MIC, the reason may be that the MAC is obtained by once approximate calculation, that is, the maximal number of grids is determined manually, while the MIC is obtained by twice approximate calculations, that is, the mutual information is calculated approximately and the maximal number of grids is determined manually. The MAC method is more robust than the MIC method, which is verified in section 3.2. Additionally, there is no ground truth of association coefficient for data with noise, which makes it difficult to estimate which of the MAC and MIC methods is accurate. It’s an unavoidable problem.

The computational complexity of the MAC and MIC methods is described as follows. In the MAC method, the K-means is used to divide each variable, and the computational complexity of K-means is *O*(*nkt*), where *n* denotes the number of samples, *k* denotes the number of clusters and *t* denotes the number of iterations, *k* and *t* are generally considered to be constants. The computational complexity of partition step is *O*(*n*). To obtain the MAC between variables, this calculation needs to call MG times, where MG = *n*^*α*^. Therefore, the computational complexity of the MAC method is *O*(*n*)*MG = *O*(*n*^*1+α*^). When the default value of α is set to 0.6, its computational complexity is *O*(*n*^*1*.*6*^). In the MIC method [21], the computational complexity of the sub-procedure OptimizeXAixs (D, Q, x) is *O*(*k*^*2*^*xy*), where *k* = *cx*, *xy* < *B* = *n*^*α*^, that is, the *O*(*k*^*2*^*xy*) = *O*(*x*^*2*^*B*). The range of *x* is from 2 to *B*/2, and the complexity of the whole MIC method is *O*(*x*^*2*^*B*) *O(*x*) = *O*(*B*^*4*^) = *O*(*n*^*4α*^). When the default value of α is 0.6, its computational complexity is *O*(*n*^*2*.*4*^).

## 6 Conclusions

In this paper, a maximal association coefficient (MAC) is proposed to detect the association strength between two variables. The idea of MAC method is to detect nonlinear association by dividing it into multiple piecewise-linear ones, which can employ computationally inexpensive Pearson coefficient to detect this nonlinear association. Since no one knows how many linear associations exist in a nonlinear association, and where are the breakpoints between two consecutive linear ones, thus partitioning method is adopted to divide the data into many different forms of grid partition. Under these grid partitions, many association coefficients can be obtained, the maximum value of them is maximal association coefficient.

The generality and equitability of the MAC have been verified by two simulation experiments, which indicates that it is reasonable to use the MAC method to detect the association strength between two variables. The research results on real data show that the MAC method can be used to detect the association strength between two variables. The information between variables mined by the MAC method may also help to complete the downstream task better in the future. The MAC between two variables can also be extended to the MAC between univariate and multivariate to explore the association between multiple variables.
